# Soft magnetic memory of silk cocoon membrane

**DOI:** 10.1038/srep29214

**Published:** 2016-07-04

**Authors:** Manas Roy, Amarish Dubey, Sushil Kumar Singh, Kalpana Bhargava, Niroj Kumar Sethy, Deepu Philip, Sabyasachi Sarkar, Alok Bajpai, Mainak Das

**Affiliations:** 1 Indian Institute of Technology Kanpur, Kanpur, Uttar Pradesh, 208016, India; 2 Defense Research Development Organization, Delhi, 110054, India

## Abstract

Silk cocoon membrane (SCM), a solid matrix of protein fiber, responds to light, heat and moisture and converts these energies to electrical signals. Essentially it exhibits photo-electric and thermo-electric properties; making it a natural electro-magnetic sensor, which may influence the pupal development. This raises the question: ‘is it only electricity?’, or ‘it also posses some kind of magnetic memory?’ This work attempted to explore the magnetic memory of SCM and confirm its soft magnetism. Fe, Co, Ni, Mn, Gd were found in SCM, in traces, through energy dispersive X-ray analysis (EDX), X-ray photoelectron spectroscopy (XPS) and inductively coupled plasma mass spectrometry (ICP-MS). Presence of iron was ascertained by electron paramagnetic resonance (EPR). In addition, EPR-spectra showed the presence of a stable pool of carbon-centric free radical in the cocoon structure. Carbon-centric free radicals behaves as a soft magnet inherently. Magnetic-Hysteresis (M-H) of SCM confirmed its soft magnetism. It can be concluded that the soft bio-magnetic feature of SCM is due to the entrapment of ferromagnetic elements in a stable pool of carbon centric radicals occurring on the super-coiled protein structure. Natural soft magnets like SCM provide us with models for developing eco-friendly, protein-based biological soft magnets.

Successful metamorphosis of dormant pupae to an adult moth is function of temperature, humidity, light, UV protection, water proofing, gaseous balance and physical protection from the predators^1^,^2^,^3^,^4^,^5^,^6^,^7^,^8^,^9^,^10^,^11^,^12^. To meet these requirements, nature evolved a ‘robust growth incubator’ in the form of ‘silk cocoon’, which is equipped with moisture, heat and light sensor^1^,^2^,^3^,^5^. Studies have confirmed that SCM converts photons, especially in UV range, to generate electricity^1^. Similarly in presence of moisture, heat energy is converted into electrical signals by SCM^2^,^3^. These electrical currents direct the growth of pupae probably by providing information about the external environment^1^.

But is it electricity alone? The pupae tends to stay inside the SCM for varying periods of 21 days to 9 months, depending on the species^1^,^5^,^12^. For the dormant pupae to survive this changing weather conditions, the ‘silk cocoon incubator’ may require an ‘*inner memory*’ so as to stabilize against the varying electrical current. *This poses the question of whether SCM has some form of magnetic elements by which it may be producing a’ soft magnetic memory’ in the presence of electric currents*? This warrants the exploration of the magnetic properties of SCM.

In this work, the magnetic properties of silk cocoon membrane of Tassar (*Antheraea mylitta*), a wild silkworm species and Mulberry (*Bombyx mori*), a widely used domesticated, commercial species of silkworm, have been studied using energy dispersive X-ray analysis (EDX), X-ray photoelectron spectroscopy (XPS), inductively coupled plasma mass spectrometry (ICP-MS), electron paramagnetic resonance (EPR) and magnetic-hysteresis (M-H).

## Results

**Figure 1:**
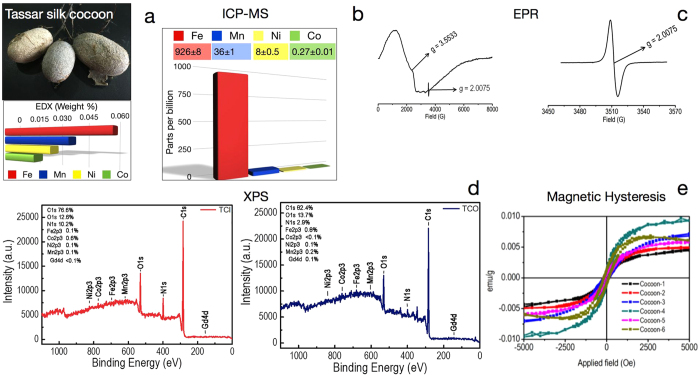
Magnetic properties of Tassar silk cocoon membrane. (**a**) Representative picture of Tassar silk cocoon. EDX analysis of SCM. (**b**) ICP-MS to quantify the elements present in the silk cocoon membrane of Tassar. The values are shown in ‘mean ± Standard deviation’. (**c**) X-band EPR of the silk cocoon at room temperature exhibit signal of a carbon radical with <g> = 2.0075 and a broad signal centered around <g> = ~3.5433 due to the presence of magnetite. The zoom in figure is provided to show carbon centric free radicals at <g> = 2.0075. (**d**) XPS analysis of the inner (plotted in red) and outer surface (plotted in blue) of silk cocoon membrane showing the presence of Fe, Mn, Ni, Co and Gd. (**e**) M-H loops of the six different cocoons measured at room temperature. The statistical analysis has been carried out from the data obtained from six randomly selected cocoons.

**Figure 2:**
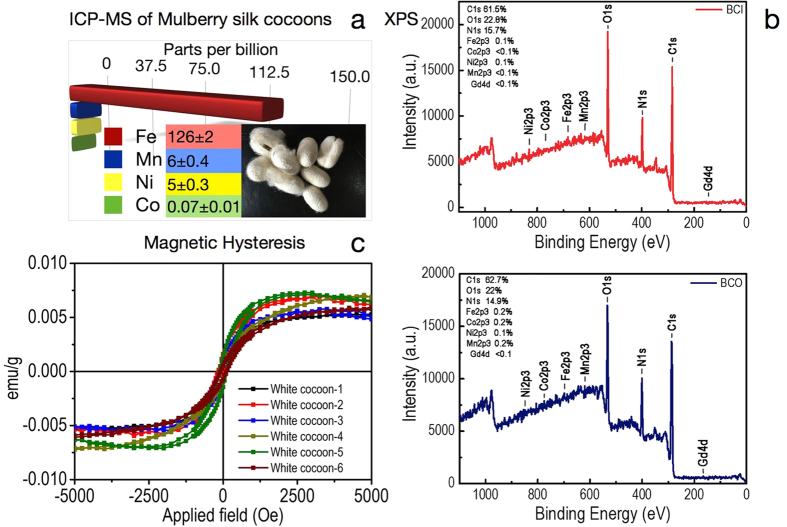
Magnetic properties of Mulberry silk cocoon membrane. (**a**) ICP-MS of mulberry silk cocoon to quantify ferromagnetic elements (Fe, Mn, Ni, Co). The inset showing the mulberry silk cocoons. (**b**) XPS spectra of inner (plotted in red) and outer (plotted in blue) surface of silk cocoon membrane. (**c**) M-H loops of the six different cocoons measured at room temperature. The statistical analysis has been carried out from the data obtained from six randomly selected cocoons.

The results section has been divided into two parts. In part 1, the results from wild silk cocoon viz., Tassar (*Antheraea mylitta*) has been discussed (Fig. 1). In part 2, the results from domestic silk cocoon viz., Mulberry (*Bombyx mori*) has been documented (Fig. 2).

Energy-dispersive X-ray spectroscopy (EDX) analysis of SCM (n = 12) confirmed the presence of four soft ferromagnetic elements (Iron; 0.06 ± 0.01 > Manganese; 0.04 ± 0.01 > Nickel; 0.03 ± 0.01 > Cobalt; 0.02 ± 0.01: mean weight percentage ± standard error) in trace amounts in tassar cocoon samples. Among all the ferromagnetic elements, concentration of iron was found to be the maximum (Fig. 1a). The ICP-MS data further validated and quantified the concentration of the four magnetic elements and were found in the following decreasing order of concentrations; Fe > Mn > Ni > Co (Fig. 1b). In the EPR spectrum, a broad resonance, centered at a “g” value of 3.5433, was observed. This was attributed to magnetite (Fe_3_O_4_). In addition, a distinct signal was observed at a “g” value of 2.0075. This corresponds to a pool of stable carbon centric free radicals. Such type of carbon-centric free radicals are very stable in nature and extremely difficult to reduce or oxidize. Anthropogenic multi-walled carbon nanotubes trapped in spider web and antennae of the silk moth preserves such pool of carbon-centered free-radicals for a long period of time and exhibit soft magnetic behavior^13^,^14^. Thus EPR measurements indicate the presence of soft ferromagnetic elements and a pool of carbon-centered free radicals in the cocoon membrane (Fig. 1c). Further a non-destructive quantification of the inner and the outer surface of silk cocoon membrane was performed using XPS. The outer surface had higher percentage of Fe as compared to the inner surface. Other three elements, Mn, Ni, Co were more or less similar in both inner and outer surface. A trace amount of Gd was observed both in the inner and the outer surfaces of silk cocoon membrane. The presence of oxygen further demonstrate that these elements are present in their oxide states (Fig. 1d). Magnetic properties of cocoon powder are measured by plotting an M-H (magnetic hysteresis) curve, as shown in Fig. 1e. The magnetic measurements were done at 300 K. The average value of saturation magnetization (M_s_) of six different silk cocoon at 5000 Oe is 0.0062 ± 0.0017 emu/g, n = 6. All the results are expressed as mean ± standard error, n = number of cocoon. The average coercivity (H_c_), a measure of the resistance to demagnetization is observed to be 127.3 ± 29.16 Oe, n = 6. The low coercivity indicates that cocoon has a soft magnetic property and it quickly gets magnetized and demagnetized as a function of applied magnetic field. The average value of remnant magnetization (M_R_) of the silk cocoon is found to be 0.0007 ± 0.0002 emu/g, n = 6.

In the subsequent paragraph, the results from domestic silk cocoon viz., Mulberry (*Bombyx mori*) has been documented. The ICP-MS results indicated the presence of Fe > Mn > Ni > Co, in a decreasing order of concentrations (Fig. 2a). The amount of the elements present in this domesticated species is much less, as compared to the wild species viz., tassar. This is partly due to the domestication process. The mulberry silkworm feed on cultivated mulberry leaves and through generations of domestication and breeding, the farmers have ensured to reduce the mineral content in the silk. Lesser mineral content, helps is smooth reeling of the silk thread. On the contrary, reeling of the silk thread in the wild silk species, is a challenging task, thus reducing its commercial viability. XPS analysis of the inner and the outer surface of mulberry silk cocoon revealed the presence of all the five magnetic elements, as observed earlier in the tassar silk viz., Fe, Mn, Ni, Co, Gd. Here too, iron was present in higher concentrations on the outer surface of the cocoon membrane. The presence of oxygen in the XPS spectra, further validates the existence of these magnetic elements in their oxide forms (Fig. 2b). The magnetic measurements were performed at 300 K. The average value of saturation magnetization (Ms) of six different mulberry silk cocoon at 5000 Oe is 0.0059 ± 0.0008 emu/g, n = 6. All the results are expressed as mean ± standard error, where n = number of cocoon. The average coercivity (Hc), was observed at 112.6 ± 8.9 Oe, n = 6. The average value of remnant magnetization (MR) of the silk cocoon was around 0.0006 ± 0.0003 emu/g, n = 6. All the values were found to be lower than the tassar cocoon (Fig. 2c). This could be explained by the fact that, all the magnetic elements are present in lesser concentrations in mulberry as compared to tassar.

## Discussion

**Figure 3:**
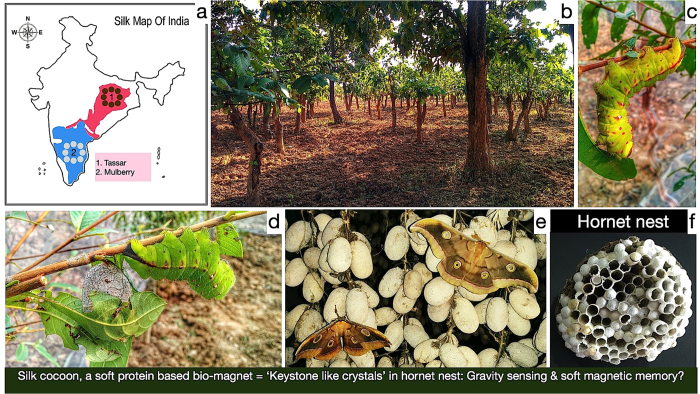
Possible origin of ferromagnetic elements in silk cocoon and their physiological significance. (**a**) Silk map of India, showing the sources of cocoons used in this study and further the geological significance of these regions. The tassar cocoons are growing in the wild of Chota Nagpur and Deccan plateau. These regions are store house of wide range of minerals. Mulberry is a domesticated species grown in parts of Deccan plateau and in the stae of Karnataka in South India. (**b**) The forest near Ranchi, Jharkhand; a place in Chota Nagpur plateau, from where the tassar silk cocoon has been obtained. The vegetation in these regions takes up the minerals present in the soil. (**c**) The possible origin of the ferromagnetic elements in silk cocoon fiber is the plant leaves, which are consumed by the growing larvae before secreting the silky fluid to form the cocoon. (**d**) A growing larvae and the tassar silk cocoon. (**e**) Adult silk moth ready to lay eggs, sitting on top of tassar silk cocoons. (**f**) Two examples of ‘insect housing’, exhibiting magnetic features and their possible role in ‘gravity sensing’ and ‘soft magnetic memory’.

This study raised the following questions:

What is the origin of ferromagnetic components in silk cocoon? (Fig. 3a–e).

How to identify the existence of the ferromagnetic elements as the form of their oxides in silk cocoon membrane? Why EPR spectra only give signature of iron and not of cobalt, nickel and manganese?

What is the origin of stable carbon-centric radical pool in SCM?

Is soft magnetic property of SCM is analogous to ‘keystones’ like structures present in the comb cells of social wasp (Vespinae) and hornet nest^15^,^16^. (Fig. 3f).

In the subsequent paragraphs, the above questions have been discussed.

In Fig. 3a, the broad silk map of India has been depicted. The wild tassar silkworm cocoon (*Antheraea mylitta* Drury) were collected from the state of Chhattisgarh and Jharkhand in the Deccan plateau of central India and Chota-Nagpur plateau regions respectively. Domesticated mulberry cocoon (*Bombyx mori*) were collected from the state of Karnataka, located in the southern part of India, which is also a part of Deccan plateau. The Deccan and Chota-Nagpur plateau regions are store house of wide range of minerals viz., mica, bauxite, copper, limestone, iron ore, monazite (a rare earth phosphate mineral) and coal to name a few. The vegetation growing in these regions have high mineral contents (Fig. 3b). The high concentration of ferromagnetic elements in the tassar silk as compared to the mulberry is possibly due to the fact that most of these silk are grown in the forest of Deccan and Chota-Nagpur plateau^5^. On the other hand, mulberry is a domesticated species and grown in controlled environment, exclusively on mulberry leaves diet. Apart from Fe, Mn, Ni and Co; Gd has been observed in the XPS spectra of silk cocoon membrane. Gadolinium (Gd) is present in the monazite sand of the Chota-Nagpur terrain as well as in the Deccan traps of southern part of India, and may amply justify its transport in the salivary gland by silk larvae in crafting cocoon, via the plant kingdom (Fig. 3c–e). The uptake of Gd^3+^ ion in plant kingdom is due to its high charge density and similar ionic radius to Ca^2+^, by mainly modulating the calcium channel and phosphate uptake. Being a strong magnetic material (in the family of lanthanides), its very presence in trace amounts, along with Fe, Mn, Ni, Co; further contribute to the magnetic properties of the silk cocoon shell. Thus silk cocoon may act as an indirect detector to trace the presence of rare and important metals in the nearby unexplored land mass. Another interesting aspect which was revealed in the XPS analysis, is the asymmetric distribution of Fe; having more Fe at the outer surface as compared to the inner surface; a similar pattern was observed in both the cocoon types. Broadly speaking, it is apparent that the cocoon membrane, somehow create a filter, for the passage of relatively stronger magnetic materials like Fe, which may adversely affect the developmental metamorphosis of the insect. This asymmetric distribution of magnetic elements across the cocoon surface needs further investigation.

The existence of these metals in oxide form is generally accepted by the inherent chemistry of cobalt, manganese, iron, nickel and also gadolinium salts. If one assumes that these are available in the cocoon from aerosol and from the larval secretion of the insect, then in both the cases, it will be prudent to consider the ready available water soluble salt of these metals and their slow transformation under environment. Under ambient pH (~7), the common metal salts get hydrolyzed to hydroxide and on aging slowly changes to oxide form. In addition for cobalt, manganese and iron, the aerial oxygen slowly oxidize these oxide to more stable mixed oxide forms, like {CoO, Co_2_O_3_=Co_3_O_4_}, {MnO, Mn_2_O_3_=Mn_3_O_4_} or {FeO, Fe_2_O_3_=Fe_3_O_4_}, the corresponding Ni could be in the state of NiO as oxidation of Ni(II) is difficult. For gadolinium the oxide form is the natural source. The oxide form can be properly indexed by using XRD pattern. But such low concentrations, did not permit to extract the diffraction pattern without noise signal. Further from XPS spectroscopy, the change in binding energy could have been correlated with the oxidation states, but here also the low concentration prevented to de-convolute the spectra without noise, to get the oxidation status of these metal oxides.

Fe, Co and Ni are ferromagnetic and are susceptible to provide EPR spectra under favoured condition. Among all the three metal oxides, the concentration of iron oxide is maximum. Here normally NiO, when contaminated with little higher oxide with Ni(III) then only one can get the EPR signature from mixed nickel oxide source. Ni(III) species can only be made at highly oxidising and high temperature condition and such environment is possibly missing during the formation of SCM. Thus in the EPR measurements no significant contribution from Ni oxide was observed. The cobalt concentration is extremely low and even if it is present there, it may have got hidden under the envelope of iron oxide signal, as its intensity will be low. Similarly under the exposure of air, if there is any Mn(II) in the form of MnO, it will get partially oxidized to Mn(III) to create Mn_2_O_3_. Such mixed valence manganese should show a broad EPR but the very characteristic six line spectral feature associated with Mn(II) cannot be missed out. EPR signal showing such hyperfine structure was not observed in the spectrum.

It is difficult to pinpoint the exact organic moiety in the silk fiber structure of cocoon, which possibly exhibits carbon centric free radical features. Since silk thread in cocoon is a super-coiled structure forming a rigid matrix, it may lead to ‘frustrated spin’ in several carbon moieties and such ‘frustrated spin on carbon’ leads to ‘carbon centric free radicals’^13^,^14^.

The stable carbon-centric free radical structure in SCM, along with the soft magnetic field generated by ferromagnetic elements, possibly serves the orientation for cellular organization and subsequent growth of larvae. Similar role of keystone like structure has been proposed for orientation to gravity in comb cells of social wasp (Vespinae) and hornet’s nest (Fig. 3f)^15^,^16^.

The presence of several magnetic elements in the silk cocoon shells, may raise an interesting issue. It highlights, how in nature, the minerals which are taken up by the plant kingdom, gets transmitted to the insect world and eventually become part of the salivary protein secretion in the form of silk. The salivary silk proteins, embedded with these magnetic ions, may constitute hitherto unknown class of ‘metal-silk protein complexes’, with the possibility of diverse magnetism; that may lead to the tailor-made development of bio-inspired soft magnetic proteins, using the combination of transition and lanthanide metal ions composite.

In summary, nature-lovers would appreciate that ‘mother *nature has developed its soft magnets by doping ferromagnetic elements in a super-coiled matrix of long carbon chain biomolecules like proteins’*; indeed an inspiration to develop ‘bio-inspired-magnets’ for memory storage devices and bio-computing.

## Methods

### Cocoon collection

Wild Tassar silkworm cocoon (*Antheraea mylitta* Drury) were collected from the state of Chhattisgarh and Jhakhand in the Deccan Plateau of Central India and Chota Nagpur plateau regions respectively. Domesticated Mulberry cocoon (*Bombyx mori*) were collected from the state of Karnataka located in the southern part of India. IPhone 6S was used for taking the photographs and were from personal collection of MD.

### Sample preparation

The collected cocoons were stored in a dust free chamber maintained at an ambient temperature for further use. For EDX, ICP-MS, XPS, EPR and VSM analysis, the cocoons were cut open and the pupae were removed. Then the cocoons were cut into small pieces and grinded to form powder. These powdered samples were used for EPR and VSM analysis. For EDX analysis, the small pieces of cocoon were coated with gold and imaged in a SEM (Scanning electron microscopy) and simultaneously EDX measurements were carried out using Supra 40 VP field emission scanning electron microscope model (Carl Zeiss NTS GmbH, Oberkochen, Germany) equipped with EDAX facility.

### Inductively coupled plasma mass spectrometry (ICP-MS)

The instrument which was used for ICP-MS was from Thermo Scientifi, Modelc X Series 2, ICP-MS. Samples for prepared using the following protocol. Tassar and mulberry cocoons with equal surface area were taken with the weight of 0.370 g and 0.960 g respectively. These cocoons were immersed in 10 ml of 69% HNO_3_ for 24 hour. After that the solution was maintained at 2% HNO_3_ by addition of distilled water. Following this step, the solution was filtered using 0.45 μ filter. The filtered samples are used for analysis following calibration with multi-standard probes.

### X-ray photo-electron spectroscopy (XPS)

XPS with auger electron spectroscopy (AES) was performed using PHI 5000 Versa Prob II,FEI Inc.

### Electron paramagnetic resonance (EPR)

Electron paramagnetic resonance (EPR) spectroscopic analysis of the silk cocoon was performed using Bruker EMX EPR Spectrometer at microwave frequency of 9.8 GHz and at 0.200 mW microwave power at 300 K.

### Vibrating sample magnetometer (VSM) studies

ADE, EV7 model of vibrating sample magnetometer (VSM) was used for studying the magnetic properties of cocoon powder, measured by plotting an M-H (magnetic hysteresis) curve. The magnetic measurements were done at 300 K.

## Additional Information

**How to cite this article**: Roy, M. *et al*. Soft magnetic memory of silk cocoon membrane. *Sci. Rep.* **6**, 29214; doi: 10.1038/srep29214 (2016).

## References

[b1] Brindan T. . The role of photo-electric properties of silk cocoon membrane in pupal metamorphosis: A natural solar cell. Sci Rep. 6, 21915, 10.1038/srep21915 (2016). 26907586 PMC4764832

[b2] Brindan T. . Electricity from the silk cocoon membrane. Sci Rep. 4, 5434, 10.1038/srep05434 (2014) 24961354 PMC4069722

[b3] Brindan T. . Harvesting electricity from human hair. J Cosmet Sci. 67, 21–36 (2016). 27319058

[b4] Kusurkar T. S. . Fluorescent silk cocoon creating fluorescent diatom using a “Water glass-fluorophore ferry”. Sci. Rep. 3, 3290, 10.1038/srep03290 (2013). 24256845 PMC3836033

[b5] Roy M. . Carbondioxide gating in silk cocoon. Biointerphases 7, 1–11 (2012). 22791361 10.1007/s13758-012-0045-7

[b6] Kirshboim S. & Ishay J. S. Silk produced by hornets: thermophotovoltaic properties—a review. Comp. Biochem. Physiol., Part A Mol. Integr. Physiol. 127, 1–20 (2000). 10.1016/s1095-6433(00)00237-3 10996813

[b7] Ishay J. S. & Barenholz-Paniry V. Thermoelectric effect in hornet (*Vespa orientalis*) silk and thermoregulation in a hornet’s nest. J. Insect. Physiol. 41, 753–759 (1995).

[b8] Horrocks N. P., Vollrath F. & Dicko C. The silkmoth cocoon as humidity trap and waterproof barrier. Comp Biochem Phys A 164, 645–652 (2013). 10.1016/j.cbpa.2013.01.023 23388210

[b9] Kaur J. . Photo-protection by silk cocoons. Biomacromolecules 14, 3660–3667 (2013). 24000973 10.1021/bm401023h

[b10] Zhang J., Rajkhowa R., Li J., Liu X. & Wang X. Silkworm cocoon as natural material and structure for thermal insulation. Mater Design 49, 842–849 (2013).

[b11] Zhang J. . Mechanical properties and structure of silkworm cocoons: a comparative study of *Bombyx mori*, *Antheraea assamensis*, *Antheraea pernyi* and *Antheraea mylitta* silkworm cocoons. Mater Sci Eng C Mater Biol Appl. 33, 3206–3213 (2013). 23706202 10.1016/j.msec.2013.03.051

[b12] Trouvelot L. The American silk worm. Am. Nat. 1, 30–38 (1867).

[b13] Sonkar S. K., Tripathi K. M. & Sarkar S. Ferromagnetic behaviour of anthropogenic multi-walled carbon nanotubes trapped in spider web indoor. J Nanosci Nanotechnol. 14, 2532–2538 (2014). 24745259 10.1166/jnn.2014.8524

[b14] Roy M. . Presence of stable carbon centric free radicals and ferromagnetic elements in the antennae and the wings of nocturnal silk moth: A magnetic nanostructure for magneto sensing. Mater. Express. 3, 43–50 (2013).

[b15] Ishay J. S. . Gravity orientation in social wasp comb cells (Vespinae) and the possible role of embedded minerals. Naturwissenschaften. 95, 333–342 (2008). 18087684 10.1007/s00114-007-0334-z

[b16] Stokroos I., Litinetsky L., van der Want J. J. & Ishay J. S. Magnetic minerals. Keystone-like crystals in cells of hornet combs. Nature. 411, 654 (2001). 11395756 10.1038/35079679

